# Body Composition and Cognitive Functioning in a Sample of Active Elders

**DOI:** 10.3389/fpsyg.2019.01569

**Published:** 2019-07-18

**Authors:** Miriam Crespillo-Jurado, Joaquín Delgado-Giralt, Rafael Enrique Reigal, Antonio Rosado, Agustín Wallace-Ruiz, Rocío Juárez-Ruiz de Mier, Verónica Morales-Sánchez, Juan Pablo Morillo-Baro, Antonio Hernández-Mendo

**Affiliations:** ^1^University of Málaga, Málaga, Spain; ^2^Consejería de Educación, Cultura y Deporte, Seville, Spain; ^3^Faculty of Human Motricity, University of Lisbon, Dafundo, Portugal; ^4^Department of Psychobiology and Methodology of Behavioral Sciences, Faculty of Psychology, University of Málaga, Málaga, Spain; ^5^Departamento de Psicología Evolutiva y de la Educación, Universidad de Málaga, Málaga, Spain; ^6^Departamento Psicología Social, Trabajo Social, Antropología Social y Estudios de Asia Oriental, Universidad de Málaga, Malaga, Spain

**Keywords:** physical activity, body composition, elderly, attention, cognitive development

## Abstract

The purpose of this paper was to analyze the relationship between body composition and cognitive functioning in an elderly people’s sample. A total of 106 older adults between the ages of 60 and 79 were involved in physical activity (*M* = 67.57; SD = 4.96). About 31.10% were men (*n* = 33) and 68.90% were women (*n* = 73). The instruments used to assess cognitive capacity were the Trail Making Test (forms A and B), the Stroop Test, and the Attention Test d2. The body composition of the participants was evaluated by electrical bioimpedance. Correlation analysis, linear regression (successive steps), and cluster analysis were carried out to analyze the relationships between the different measures. The results showed significant relationships between the analyzed variables. In addition, muscle mass predicted the cognitive functioning values. These results suggest that healthy lifestyles, including physical activity, are essential for well-being and quality of life in older people. In addition, it appears from the results found that it would be necessary for these lifestyles to contribute to preserving their level of physical condition, because of the possible impact it would have on their health.

## Introduction

Active aging is associated with positive health effects, improved psychosocial functioning, and lower mortality risk (Haskell et al., 2007; García-Molina et al., 2010; Reigal and Hernández-Mendo, 2014). Specifically, regular physical activity is associated with improvements in the cognitive functioning of older people, as evidenced in research conducted in recent years, in which positive relationships have been found with abilities such as attention, memory or executive functioning (Bherer, 2015; Daly et al., 2015; Iuliano et al., 2015; Sánchez et al., 2018).

Specifically, attentional capacity has been studied from numerous authors (Marshall et al., 2015; Strait et al., 2015). This is a basic cognitive capacity that is related to multiple cognitive processes such as memory, executive control, language or learning, and has a great interest in educational or clinical field (Maureira and Flores, 2017). Dimensions such as selective attention is considered as an indicator of the ability to attend to specific stimuli and ignore others, being a relevant process to successfully perform a broad set of tasks that allow people to adapt correctly to their environment (Estévez-González et al., 1997; Giuliano et al., 2014).

Likewise, executive functioning refers to a set of complex cognitive processes considered relevant to people’s functioning (Davis et al., 2011; Kramer and Colcombe, 2018) and is related to the control of thought and behavior (Zelazo and Carlson, 2012). These processes include aspects such as inhibitory control, cognitive flexibility, the organization and planning tasks, the selection of objectives and the establishment of strategies to achieve them, or working memory (Banich, 2009; Barkley, 2012; Diamond, 2013; Karr et al., 2018).

These functions may suffer a natural decline throughout the aging process (Schiebener and Brand, 2017), being the main cause of loss of autonomy in the elderly population (Boucard et al., 2012; Weinstein et al., 2012). Therefore, the main lines of research encourage cognitive assessment in adulthood as an effective predictor of cognitive decline in old age (Ferreira et al., 2015), as well as promoting healthy and active lifestyles (Ballesteros et al., 2013).

Although physical activity has been described as a predictor of cognitive functioning, several studies have highlighted the need to assess the impact it has on physical condition to adequately interpret its incidence on brain development (Reiter et al., 2015). In other words, physical activity must modify physical and physiological parameters to modulate the impact on health. Thus, several studies have revealed that the benefits of physical activity on cognitive functioning in the elderly were preceded by changes in physical condition and brain structure (Guzmán-Cortés et al., 2015; Fernandes et al., 2017; Jonasson et al., 2017).

Cognitive aging, and its relationship to physical condition and body composition, has been the subject of several studies (Cansino et al., 2011; Albinet et al., 2012; Weinstein et al., 2012; Müller et al., 2017). Some studies have shown that cardiovascular fitness, achieved through moderate to vigorous physical activity, has been associated with both relative preservation of inhibitory capacity later in life and general improvements in cognitive functions throughout adulthood (Kamijo et al., 2009; Boucard et al., 2012; Kerr et al., 2013). In addition, it has been shown in several studies that the organic deterioration produced by aging could be cushioned by physical condition and body composition, which are predictors of some health parameters. Thus, a study of 32 Parkinson’s patients investigated posture, stability, and body composition as relevant factors from a neurological disease prevention perspective (Wilczyński et al., 2017).

Similarly, at present, there is a special interest in structuring healthy lifestyles and the practice of physical activity in those cases in which it is presented as an alternative to pharmacological interventions, showing potent improvements in executive control processes in women at risk of cognitive impairment (Baker et al., 2010). In response to this demand, new lines of research on the cognitive benefits associated with physical activity place their attention not only in the benefits derived from the practice of aerobic activities by itself but also in programs that also require cognitive execution (Reyna et al., 2013; Diamond, 2015). The results obtained by these studies provide new data on the positive impact of physical activity on the cognitive health of older people and open the door to a new range of work pathways (Reigal and Hernández-Mendo, 2014).

Thus, the existing literature has shown that only practicing physical activity does not ensure improvements in health, but it is necessary to observe the impact it has on the organism. For this reason, to analyze whether there are significant differences between people who carry out physical activity by differentiating two groups in terms of a better body composition condition, the aim of this work was to evaluate the relationships between body composition and cognitive functioning in a sample of active elderly people in the province of Malaga.

## Materials and Methods

### Participants

In total, 106 active older participants (age: *M* = 67.57; SD = 4.96) from the municipalities of Benalmádena (*n* = 36) and Ronda (*n* = 70), both in the province of Málaga (Spain), were evaluated. All engaged in regular physical activity, specifically maintenance activities in organized groups with a frequency of 2 days a week for 60 min. About 31.10% were men (*n* = 33) and 68.90% were women (*n* = 73) and had a highly variable practical experience, ranging from 1 to 46 years (*M* = 9.19; SD = 9.37).

### Measurements

#### Cognitive Flexibility

As a measure of executive function, cognitive flexibility includes the capacity for cognitive control and implies the capacity to inhibit the first response, the automatic response or the most obvious response, flexibly changing the choices to give way to a second response pertinent to the variation of requirements of the task (Buller, 2010). To obtain a measurement of the cognitive flexibility of the participants, the Trail Making Test was used in its forms A and B (Reitan, 1958, 1992; Tombaugh, 2004). The Trail Making Test Form A consists of 25 numbers distributed randomly on a sheet of paper, which must be connected by a line in ascending order by the participants, leaving no unconnected numbers. On the other hand, the Trail Making Test Form B requires the participant to alternate numbers and letters by joining upwards and interspersed 13 numbers with the first 12 letters of the alphabet. Prior to each form of the test, the participant has a detailed example, which allows the researcher to make sure that the participant has understood the instructions correctly. For both form A and form B of the test, the performance of the tests is timed by the assessor.

#### Inhibitory Control

Measurement of executive function involves the selection of relevant stimuli required for the achievement of the objective, the maintenance of focus on relevant stimuli, and a low level of sensitivity to interference by irrelevant stimuli (Buller, 2010). For the evaluation of inhibitory control, the Stroop Test was used (Stroop, 1935; Golden, 2001). In this test, the participant must do three consecutive parts, having 45 s to accomplish each of them. Thus, the first of the tests presents the words “RED,” “GREEN,” and “BLUE” distributed along a list of 100 words, asking the participant to name as many as possible in the established time. The second of the tests presents the text “XXXX” written in green, red or blue, configuring in total a new list of 100 elements, in this case, the participant must indicate the color of the ink in which it is written. In the third and last test, 100 words are presented that indicate the colors “RED,” “GREEN,” and “BLUE” but written in a different one, which generates an interference effect. The participant, as in the second test, is urged to say the color of the ink in which the words are printed, thus inhibiting the direct reading of them.

#### Attention

For the evaluation of the participants’ attention, the Attention Test d2 was used (Brickenkamp and Zillmer, 2002). This test consists of a total of 14 lines, each of which contains the letter d repeatedly, as well as the p interspersed. The task asked the subject to mark each letter d that has two small lines distributed either above the letter, below or in both places, adding in any case a total of two lines around the letter. To do this, the subject has 20 s to make as many marks as possible in each line, once this time has passed, they must continue the task on the next line. The main concepts to be evaluated by the test are those of sustained attention, requiring the participant to maintain continuous attention for approximately 10 min; and selective attention, with the participant having to select only those relevant stimuli from each line, inhibiting the rest of the distracting stimuli.

#### Anthropometric Measurements and Body Composition

To analyze the height, a tape measure was used and placed on the wall completely straight and glued to prevent displacement during the measurement and a square was placed over the head of the participant, noting the result later. The researchers read about it beforehand so that the measurements would be as realistic as possible with the means available. In addition, a bioimpedanciometer (Tanita® Body Composition model BC-601) was used. The model is a tetra-polar machine, which has electrodes in four points of contact on the sole of the foot, and two on a support that is grasped with the hands, which use a low frequency signal to obtain the measurement to evaluate corporal fat, percentage of muscular mass, and the percentage of bone mass. Prior to obtaining the data, parameters such as age, gender, the person’s level of physical activity and height are added.

### Procedure

Prior to carrying out the study, directors and monitors of the Benalmádena and Ronda Municipal Sports Center were contacted in order to obtain their consent, subsequently the participants were informed about the study that was going to take place, the aspects relating to their privacy and voluntary nature of their collaboration. In addition, prior authorization was obtained from the Ethics Committee of the University of Malaga (no. 244, CEUMA Registry No.: 19-2015-H). After the first contact, the volunteer participants were summoned individually in offices set up for the interviews and informed consent of each participant was obtained before starting the evaluations. Also, ethical principles of the Declaration of Helsinki were respected (World Medical Association, 2013).

Each evaluation session lasted approximately 30–60 min, although for personal reasons of the participants, with some of them it was necessary to have more than one appointment to perform all the tests. The structure of the sessions was as follows: presentation of the study, obtaining informed consent, brief initial interview to obtain health-related data such as drug consumption, cognitive tests, height measurement, and obtaining anthropometric measurements and body composition by bioimpedance. Most of the measurements were intended to be taken in the morning, without shoes and with comfortable, light clothes. Considering aspects such as not attending after a meal or intense exercise as indicated by the device’s instructions, and after a brief period of rest seated before.

### Data Analysis

Descriptive and inferential analysis of the data was performed, as well as a study of data normality (Kolmogorov-Smirnov and Shapiro-Wilks). Correlations between physical and cognitive measures were analyzed using Pearson’s bivariate coefficient. Previously, a treatment of lost values was carried out. In addition, a *k*-means cluster analysis was performed and two groups were generated. The U-Mann Whitney test was used to compare scores between groups. The *p* < 0.05 was considered for rejecting null hypothesis. The statistical package used was the SPSS version 20.0.

## Results

### Descriptive and Correlation Analysis

Table 1 shows the data referring to the descriptive statistics of maximum values, minimum values, mean values, and standard deviation of the variables studied. Normality analysis was performed using the Kolmogorov-Smirnov test, as well as analysis of asymmetry and kurtosis. In the variables where the criterion of normality was not fulfilled, a transformation of the score with ln(*x*) was carried out, obtaining proportional distributions that satisfactorily fulfilled this criterion (Commissions-D2: *Z* = 1.18, *p* > 0.05; total effectiveness of the test (TOT)-D2: *Z* = 1.35, *p* > 0.05; Trail Making Test A: *Z* = 0.69, *p* > 0.05).

**Table 1:** Descriptive and normal statistics.

	Mean	SD	Asymmetry	Curtosis	Z
Age	67.57	4.96	0.26	−0.75	0.89
% Fat mass	37.54	6.42	0.12	0.66	0.76
% Muscle mass	59.23	6.65	0.04	1.35	0.93
% Bone mass	3.19	0.33	0.18	1.15	0.88
Physical practice experience (years)	9.19	9.37	1.72	3.04	10.80**
D2-TR	41.15	12.68	0.80	2.71	1.09
D2-TA	38.59	14.31	0.88	2.27	1.34
D2-O	43.45	21.29	0.58	0.15	1.06
D2-C	24.13	16.92	0.87	0.18	1.44*
D2-TOT	36.25	11.63	0.45	0.24	10.81**
D2-CON	31.52	14.45	−0.01	−0.52	1.04
D2-TR+	41.15	11.86	−0.28	0.78	0.68
D2-TR−	48.85	10.42	0.53	−1.04	1.30
D2-VAR	50.94	22.22	0.21	−0.52	0.79
Trail Making Test A	48.31	19.18	1.14	1.86	1.41*
Trail Making Test B	119.85	52.56	0.95	1.05	1.25
Stroop-words	88.81	20.42	0.31	−0.33	0.57
Stroop-colors	60.72	13.17	0.20	−0.11	0.71
Stroop-words/colors	34.81	12.71	0.88	1.01	0.84

*Z = Kolmogorov-Smirnov; D2-TR = total number of attempts; D2-TA = total hits; D2-O = omissions; D2-C = commission; D2-TR+ = line with the highest number of attempted elements; D2-TR− = line with fewer items attempted; D2-TOT = total effectiveness in the test; D2-CON = concentration index; D2-VAR = variation index*.

* p < 0.05;

** *p < 0.01*.

Table 2 shows the results of Pearson’s correlation analysis. This analysis indicated the existence of relationships between body composition and cognitive measures. Specifically, the most remarkable relationships occurred with the TOT, CON and TR+ measurements of Test D2, as well as with the TMT measurements (forms A and B). Age was only significantly associated with TR+ (D2) and TMT (forms A and B).

**Table 2:** Correlation level (Pearson).

	D2									Trail Making Test		Stroop		
	TR	TA	O	C	TOT	CON	TR+	TR−	VAR	A	B	W	C	W/C
Age	−0.16	0.6	0.11	−0.04	−0.13	−0.06	−0.21*	−0.05	−0.15	0.34**	0.25*	−0.07	−0.03	0.00
% FM	−0.18	−0.16	−0.2	−0.11	−0.24*	−0.21*	−0.10	−0.22*	0.05	0.28**	0.27**	−0.10	−0.10	−0.14
% MM	0.23*	0.19	0.00	0.09	0.29**	0.25**	0.13	0.23*	−0.04	−0.31**	−0.32***	0.11	0.15	0.21*
% BM	0.20*	0.18	0.01	0.06	0.26**	0.22*	0.13	0.21*	−0.01	−0.28**	−0.27**	0.09	0.12	0.19*
PPE	−0.03	−0.17	−0.14	−0.01	−0.07	−0.09	0.08	−0.16	0.19	−0.10	−0.11	0.15	0.06	0.05

*FM = fat mass; MM = muscle mass; BM = bone mass; PPE = physical practice experience; D2-TR = total number of attempts; D2-TA = total hits; D2-O = omissions; D2-C = commission; D2-TR+ = line with the highest number of attempted elements; D2-TR− = line with fewer items attempted; D2-TOT = total effectiveness in the test; D2-CON = concentration index; D2-VAR = variation index; W = words; C = colors; WC = words/colors*.

* p < 0.05;

** p < 0.01;

*** *p < 0.001*.

### Linear Regression Analysis

Table 3 shows the linear regression analysis performed using the successive steps technique. The predictor variables in each model are visceral fat and muscle mass, and the criterion variables are components of the different cognitive questionnaires. This procedure has been used to check out which aspects of body composition (in this case muscle mass and visceral fat) allow predicting the value of the cognitive questionnaires (D2, Trail Making Test and Stroop). The variables excluded in each case are due to their lack of significance (*p* > 0.05).

**Table 3:** Linear regression analysis (successive steps).

Variable criterion	*R*	*R* ^2^ corrected	D-W	Predictor variables	Beta	*t*	*T*	FIV
D2-TR	0.27	0.06	2.17	% MM	0.27	2.76**	1.00	1.00
D2-TOT	0.38	0.13	1.86	% MM	0.38	3.24**	1.00	1.00
D2-CON	0.33	0.10	1.60	% MM	0.33	2.72**	1.00	1.00
TMT-A	0.40	0.15	1.89	% MM	−0.40	−4.30***	1.00	1.00
TMT-B	0.37	0.13	1.99	% MM	−0.37	−3.99***	1.00	1.00
Stroop W/C	0.21	0.04	2.03	% MM	0.21	2.23*	1.00	1.00

*D2-TR = line with fewer items attempted; D2-TOT = total effectiveness in the test; D2-CON = concentration index; TMT-A = Trail Making Test A; TMT-B = Trail Making Test B; Stroop-W/C = Stroop words/colors*.

* p < 0.05;

** p < 0.01;

*** *p < 0.001*.

Referring to the Durbin-Watson statistic, the values ranged from 1.60 to 2.17. Based on the interpretation of Pardo and Ruiz (2005), when the statistician is between 1.50 and 2.50, it can be assumed that the waste is independent and the assumption of independence of independent variables with respect to the dependent is fulfilled. For their part, collinearity statisticians indicated appropriate variance and tolerance inflation values.

As can be seen in Table 3, the analysis, using the successive steps method, indicated that the percentage of the muscle mass variable predicted the scores in some measures of the attention test d2, specifically TR (*R* = 0.27; *R^2^ adjusted* = 0.06; *F* = 7.61; *p* < 0.01), TOT (*R* = 0.38; *R^2^ adjusted* = 0.13; *F* = 10.51; *p* < 0.01), CON (*R* = 0.33; *R*^2^ *adjusted* = 0.10; *F* = 18.98; *p* < 0.001), as well as Form A scores (*R* = 0.40; *R*^2^ *adjusted* = 0.15; *F* = 18.56; *p* < 0.001) and B of the Trail Making Test (*R* = 0.37; *R*^2^ *adjusted* = 0.13; *F* = 15.91; *p* < 0.001) and Stroop P-C (*R* = 0.21; *R*^2^ *adjusted* = 0.04; *F* = 4.97; *p* < 0.05).

### *K*-Means Cluster Analysis

**Figure 1:**
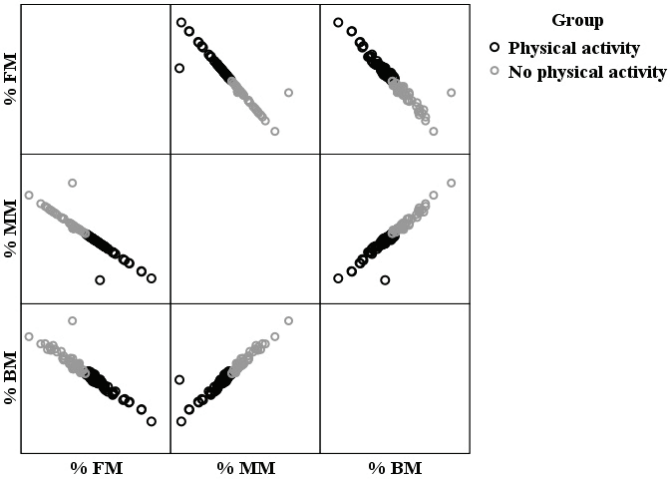
Clusters made from body composition measures. Note. FM = fat mass; MM = muscle mass; BM = bone mass.

Through cluster analysis (*k*-means), two clusters were generated depending on the variable’s fat mass percentage, muscle mass percentage, and bone mass percentage. Each case was well classified, since the maximum distance of each one from the center of its group (14.22) was less than the distance between the centers of the clusters (50.36). Thus, and as can be seen in Figure 1, the two groups constituted were characterized by (1) having a higher percentage of bone and muscle mass, as well as lower fat mass (*n* = 46; 20 men and 26 women; age: *M* = 66.76; SD = 4.72; practical experience: *M* = 8.79; SD = 10.55) and (2) have a lower percentage of bone and muscle mass, as well as higher fat mass (*n* = 60; 13 men and 47 women; age: *M* = 68.18; SD = 5.09; practical experience: *M* = 9.39; SD = 8.82). The two groups did not show differences in age (*p* < 0.05) or physical practice experience (*p* < 0.05), although they did show differences in gender distribution (*χ* ^2^ = 5.78; *p* < 0.05).

Table 4 shows the descriptive statistics of the variables analyzed and the normality tests (Kolmogorov-Smirnov and Shapiro-Wilks), for the groups formed from the level of physical condition presented. As it can be seen, group 1 had higher scores on body composition measures (*p* < 0.001), on TR, TA, TR-, TOT, and CON (d2) (*p* < 0.05) than group 2.

**Table 4:** Descriptive and normal measures of the variables analyzed as a function of physical condition.

	Group 1 (*n* = 46)					Group 2 (*n* = 60)					
	M	SD	A	K	S-W	M	SD	A	K	S-W
**Body composition**
Age	66.76	4.72	0.19	−1.10	0.94*	68.18	5.09	0.27	−0.66	0.77	
% FM	31.84***	3.81	−1.12	0.87	0.90**	41.91	4.20	1.67	2.73	1.41*	
% MM	64.91***	4.43	1.61	3.02	0.84***	54.88	4.41	−1.70	2.63	1.57*	
% BM	3.47***	0.23	1.35	2.23	0.88***	2.97	0.21	−1.47	2.51	1.28	
PPE	8.79	10.55	2.41	6.44	0.85***	9.39	8.82	1.22	0.62	1.32	
**Cognitive functioning**											
D2-TR	43.92*	13.53	1.20	4.18	0.92**	39.03	11.67	0.26	0.19	0.77	
D2-TA	42.56*	15.00	1.35	2.93	0.91**	35.55	13.09	0.32	0.67	1.10	
D2-O	44.87	18.21	0.78	0.86	0.95*	42.37	23.47	0.56	−0.15	0.96	
D2-C	24.70	18.57	0.77	−0.15	0.90*	23.77	16.00	0.96	0.58	1.14	
D2-TOT	39.58*	12.10	0.43	0.70	0.97	33.70	10.68	0.36	−0.47	1.62*	
D2-CON	35.15*	13.55	0.03	−0.52	0.97	28.73	14.62	0.05	−0.55	0.83	
D2-TR+	46.97	14.27	1.10	1.94	0.92**	40.27	11.13	0.27	0.43	1.20	
D2-TR−	46.64*	19.17	0.32	−0.40	0.98	54.23	23.94	0.02	−0.62	0.55	
D2-VAR	40.86	15.02	0.15	0.59	0.97	39.85	16.70	0.46	0.01	0.63	
TMT-A	46.67	19.38	0.67	−0.30	0.94*	49.57	19.09	1.56	3.63	1.38*	
TMT-B	113.83	47.13	0.38	−0.33	0.97	124.47	56.33	1.15	1.23	1.25	
Stroop-words	91.52	21.28	−0.05	−0.69	0.98	86.73	19.67	0.62	0.37	0.71	
Stroop-colors	61.78	13.72	0.32	0.16	0.98	59.90	12.80	0.06	−0.39	0.58	
Stroop-W/C	36.43	14.98	0.86	0.57	0.94*	33.57	10.62	0.54	0.21	0.90	

*A = asymmetry; K = Kurtosis; S-W = Shapiro-Wilk test; FM = fat mass; MM = muscle mass; BM = bone mass; PPE = physical practice experience; D2-TR = total number of attempts; D2-TA = total hits; D2-O = omissions; D2-C = commissions; D2-TOT = total effectiveness in the test; D2-CON = concentration index; D2-VAR = variation index; D2-TR+ = line with the highest number of attempted elements; D2-TR− = line with fewer items attempts; TMT-A = Trail Making Test A; TMT-B = Trail Making Test B; Stroop-W/C = Stroop words/colors*.

* p < 0.05;

** p < 0.01;

*** *p < 0.001*.

## Discussion

The aim of this work was to analyze the relationships between body composition and cognitive functioning, specifically executive and attentional functioning variables, in an active older people sample. Specifically, the aim was to explore whether, despite physical activity, there were differences depending on the impact produced on measures of physical condition and body composition. For this study, we have considered the percentage of fat, bone, and muscle masses. In addition, as complementary measures, age and experience of physical practice were valued as measures that could generate biases in the analysis realized.

The correlation and linear regression analysis carried out indicated that there were significant relationships between the body composition measures analyzed and some of the cognitive functioning measures presented in this study. However, the variables age and physical practice experience had little impact on the analysis performed, suggesting that the impact of lifestyle on the body is an essential phenomenon when exploring health relationships in people. This has already been highlighted in previous research and is considered a fundamental line of work when dealing with this object of study (Reiter et al., 2015; Fernandes et al., 2017). Therefore, the relationships between physical activity and health in older people should be analyzed in response to the changes that occur in the organism, as these data will indicate if the health is being truly improved. This matches with previous findings that had observed relationships between increased fat mass and decrement of muscle mass in the cognitive functioning of older people (Papachristou et al., 2015).

These results are interest given that regular physical exercise is one of the main, non-pharmacological strategies, to age healthier and improve quality life of older people (Kamijo et al., 2009; García-Molina et al., 2010). Although direct physical performance level has not been evaluated, muscle mass percentage is shown to be the variable that best predicts cognitive functioning in the participants group from this study. Yoon et al. (2017) had already indicated that improving strength in older people could help preserve their cognitive functioning. In this sense, physical exercise and increased physical performance would be linked to this phenomenon, helping to preserve muscle mass during the aging process. For this reason, the results found would support what was evidenced in previous studies, indicating that active lifestyles and adequate physical condition would help protect cognitive deterioration in elderly people (Baker et al., 2010; Boucard et al., 2012; Ballesteros et al., 2013; Kerr et al., 2013; Bherer, 2015; Müller et al., 2017). In addition, these body-composition indicators contribute to a better follow-up of the results of the health programs applied to older people.

In recent years, there has been a notable increase in neuroimaging studies that have corroborated neurobiological correlates, when positive influences of habitual exercise on cognitive functions have been analyzed. Although more work is still needed to give definitive answers to certain questions such as, the differentiated effects of physical exercise programs, how they interact with other elements of lifestyles like food and rest, or how the type of work done throughout life or other leisure tasks, might also contribute to preserving and improving cognitive functioning in people (Yanagisawa et al., 2010; Bherer et al., 2013). In fact, in recent years, evidence has emerged on the positive effects of physical exercise programs integrated with cognitive involvement tasks, which arise as an appropriate way to enhance the effects of isolated physical exercise on people’s cognitive abilities (Rey et al., 2011; Reyna et al., 2013; Reigal and Hernández-Mendo, 2014). Among other reasons, improving cognitive functioning at these ages is important, due to its involvement in social adaptation processes and adjustment to the environment (Kotwal et al., 2016).

For this reason, the present work presents some limitations that could be corrected in future works. On one hand, it would be interesting to extend the study sample in order to carry out more analysis based on variables such as gender or classify according to other variables specific to healthy lifestyles such as diet, type of academic training or usual rest time. On the other hand, it would be appropriate to include some physical performance measure, to have a broader vision of the physical state of those evaluated. Likewise, different programs for physical exercise could be contrasted, to observe if the integration of interaction activities and cognitive implication during the physical work could produce any differences between the participants. In addition, the bioimpedance analysis has a margin for error. Therefore, these measures could be supplemented with others that offered a greater approximation to the real value of body composition.

Despite the study limitations, the data found contribute to the body composition and physical condition research on the cognitive functioning of older people, suggesting that age and practical experience is not as relevant as the impact it has on people’s bodies. This suggests that beyond the exercise programs that are carried out, an exhaustive control of the effects on people would be necessary, with the objective of foreseeing with greater adjustment of the implications that it could have on their health. These objectives are of high social relevance, given their impact on public health and on quality life of older people, which could help them to have a more satisfying life (García-Molina et al., 2010; MacAuley et al., 2016).

## Conclusions

Data obtained in this work have pointed to positive relationships between the muscle mass percentage and cognitive functioning measures such as attention and executive functions. Also, older adults with higher muscle percentage and bone mass, as well as lower fat mass, showed better scores in attention measures. Results suggest that developing active lifestyles that promote better fitness could help preserve cognitive decline in this population.

## Data Availability

The datasets generated for this study are available on request to the corresponding author.

## Author Contributions

AH-M, VM-S, RJ, AR, RR, AW-R, and JM-B helped in design of the work and acquisition, analysis, and interpretation of data for the work. MC-J, JD-G, RR, and AH-M helped in acquisition, analysis, and interpretation of data for the work. MC-J and JD-G helped in acquisition and analysis of data for the work. All authors helped in drafting the work or revising, final approval of the version, and agreement to be accountable for all aspects of the work.

### Conflict of Interest Statement

The authors declare that the research was conducted in the absence of any commercial or financial relationships that could be construed as a potential conflict of interest.
